# Perceiving polarization with the naked eye: characterization of human polarization sensitivity

**DOI:** 10.1098/rspb.2015.0338

**Published:** 2015-07-22

**Authors:** Shelby E. Temple, Juliette E. McGregor, Camilla Miles, Laura Graham, Josie Miller, Jordan Buck, Nicholas E. Scott-Samuel, Nicholas W. Roberts

**Affiliations:** 1 School of Biological Sciences, University of Bristol, Bristol BS8 1TQ, UK; 2 School of Experimental Psychology, University of Bristol, Bristol BS8 1TQ, UK

**Keywords:** retina, Viking navigation, Henle fibre layer, carotenoid, zeaxanthin, lutein

## Abstract

Like many animals, humans are sensitive to the polarization of light. We can detect the angle of polarization using an entoptic phenomenon called Haidinger's brushes, which is mediated by dichroic carotenoids in the macula lutea. While previous studies have characterized the spectral sensitivity of Haidinger's brushes, other aspects remain unexplored. We developed a novel methodology for presenting gratings in polarization-only contrast at varying degrees of polarization in order to measure the lower limits of human polarized light detection. Participants were, on average, able to perform the task down to a threshold of 56%, with some able to go as low as 23%. This makes humans the most sensitive vertebrate tested to date. Additionally, we quantified a nonlinear relationship between presented and perceived polarization angle when an observer is presented with a rotatable polarized light field. This result confirms a previous theoretical prediction of how uniaxial corneal birefringence impacts the perception of Haidinger's brushes. The rotational dynamics of Haidinger's brushes were then used to calculate corneal retardance. We suggest that psychophysical experiments, based upon the perception of polarized light, are amenable to the production of affordable technologies for self-assessment and longitudinal monitoring of visual dysfunctions such as age-related macular degeneration.

## 1. Introduction

Polarization is another dimension of light, just like colour and intensity, which can provide distinct and useful information about a visual scene. Many animals, particularly invertebrates, are sensitive to the polarization of light and use this information for navigation, finding water, predator/prey detection and communication (reviewed in [1]). Though most of us are unaware of our capacity to do so, humans can also perceive the polarization of light. We detect the orientation of polarized light using ‘Haidinger's brushes’, an entoptic visual phenomenon described by Wilhelm Karl von Haidinger in 1844 [2]. He reported [3] that when viewing a polarized light^1^ field, with no spatial variation in intensity or colour, it was possible for someone with normal sight to perceive a faint pattern of yellow and blue bowtie-like shapes that intersect at the viewer's point of fixation (electronic supplementary material, figure S1). Haidinger's brushes can be observed by looking at a region of blue sky approximately 90° from the sun, particularly around sunset or sunrise, or by looking at a region of white on a liquid crystal display (LCD). The effect vanishes within about 5 s, but can be maintained and/or increased in salience by rotating the eye around the primary visual axis relative to the light field, e.g. tilting one's head side to side.

The mechanism mediating human polarization sensitivity is understood to be dependent on the presence of dichroic carotenoid pigments found in the macula, which have an average orientation perpendicular to the Henle fibres that radiate from the centre of the fovea [4–12]. The macular pigments, which give the macula lutea its yellow colour, have a spectral absorbance that peaks at 458 nm [11] and is the same (within the margins of experimental uncertainty) as the wavelength of maximum sensitivity for Haidinger's brushes, which peaks around 460 nm [6,11]. The role of the macula in producing Haidinger's brushes has led to investigation of the phenomenon as a potential approach for screening for central visual field dysfunction, including congenital abnormalities of the macula, some forms of colour blindness, macular edema, strabismus and amblyopia [13–15]. The correlation between low macular pigment density and the risk of developing age-related macular degeneration (AMD) [16], the leading cause of blindness in the West [17], means that polarization-based testing could potentially offer a simple and affordable means of identifying those at risk of AMD and monitoring disease progression. Several methods of measuring macular pigment density (e.g. colour matching, heterochromatic flicker photometry and threshold sensitivity) are available [18–20], but by testing the perception of Haidinger's brushes rather than just pigment density we may gain additional information about the spatial ordering of carotenoids with respect to the Henle fibre layer. The potential clinical value of this information, in terms of insight into the structural integrity of the Henle fibre layer or the fidelity of the mechanism that generates orientational order among the pigment of the macula, remains unexplored. Our first objective was to develop the technology and a methodology for testing the lower limits (threshold) of per cent polarization at which humans can still detect Haidinger's brushes. This is a step towards correlating per cent polarization threshold with an individual's macular pigment density.

Our second objective was to explore the effects of corneal birefringence on the perceived orientation of Haidinger's brushes. The corneal stroma is made up of densely packed collagen in alternating layers of parallel fibres and gives rise to intrinsic and form birefringence [21]. Polarized light propagating along the mutually perpendicular ‘fast’ and ‘slow’ axes of a birefringent material will accumulate a relative time delay or phase shift referred to as ‘retardance’. The magnitude of corneal retardance depends on both the thickness and birefringence of the stromal layers and varies between individuals [22,23], as does the orientation of the fast and slow axes with respect to the horizontal (corneal azimuth) [22]. Reports of perceptual effects caused by corneal retardance have so far been limited to perceived changes in the dichroic ratio of the retina as a function of angle of polarization [6] and the appearance of Haidinger's brushes generated by circularly polarized light [24]. However, simulations assuming a uniaxial model of corneal birefringence predict that there should be a mismatch between the angle of polarization observed and the orientation of Haidinger's brushes (perceived angle of polarization) [25]. By quantifying the predicted nonlinear rotational dynamics of Haidinger's brushes, we have been able to obtain a measure of corneal retardance and corneal azimuth for 21 individuals using a purely psychophysical approach.

## 2. Material and methods

The study was divided into two parts. In Part One, the per cent polarization threshold for detection of Haidinger's brushes was measured using a single interval binary choice paradigm. In Part Two, rotational dynamics of Haidinger's brushes were characterized using an alignment task, from which corneal retardance (corneal polarization magnitude) and corneal azimuth (a variant of corneal polarization axis) were calculated.

Twenty-seven (15 female and 12 male) students and colleagues took part in the study. Participants provided informed consent and all procedures were approved by the University of Bristol Ethics Committee (approval no. 251012838C). Participants removed prescription glasses if worn, though contact lenses were left in place and their presence recorded. Four people did not complete the qualifying/training stage (see below) for the per cent polarization threshold test, and three people did not participate in the alignment task, one of which was one of the four who did not complete the training stage and therefore did not contribute to either part of the study.

### (a) Part One: varying per cent polarization for threshold testing

**Figure 1.**
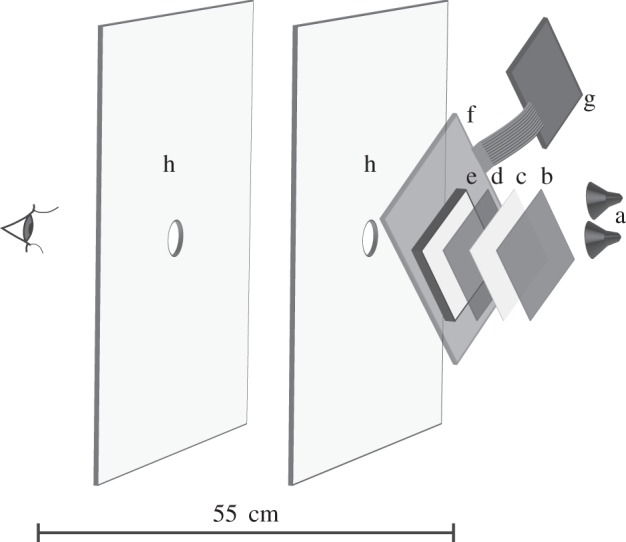
Schematic diagram of the modified LCD for presenting visual stimuli in polarization-only contrast at different per cent polarization levels. Light from two 6 W white LEDs (a) passed through a neutral density filter (b) if required (see supplementary table S1), and was then depolarized by a thin sheet of Teflon (c), before passing through a linear polarizer (d). It was then partially depolarized by one of our custom-made volume diffusers (e), after which the angle of polarization was rotated by a twisted nematic liquid crystal panel (f) from which the electronics (g) had been displaced. The various filters (b–e) were in close apposition in the actual device but are presented in an exploded format here to enable clear labelling and visualization. The image on the front of the modified LCD was positioned 55 cm from the eye of the observer and was only visible when viewed through two cardboard panels with aligned apertures (h), which limited the field of view to 5.9°.

We modified a LCD computer monitor (15 inch, Type: VPC15AS1, Viglen, St. Albans, Hertfordshire, UK) to produce stimuli that varied in polarization, but not perceptibly in intensity or wavelength (see calculation of intensity contrast and chromatic just notable differences in electronic supplementary material, table S1 and figure S2). Our modifications included removal of the front polarizer (as per [26,27]), which meant that changes in greyscale (on a normal monitor) resulted in changes in the polarization angle (up to 65°). We also removed the internal lighting, diffusers, back polarizer, a section of the back casing and relocated the electronics. This allowed fitting of our own polarizers and filters, and the passage of light from two externally mounted LED lights (Philips 6 W, Master LED spot MV GU10, Eindhoven, NL) (figure 1), allowing us to vary per cent polarization.

We constructed a set of solid, 4 mm thick, volume diffusers in which scattering material partially depolarized the light. We varied the density of scattering material incorporated into the volume diffusers to produce filters at a range of per cent polarization values (electronic supplementary material, table S1). Complete measurements of the optical properties are reported in electronic supplementary material, table S1 and figure S3, and filters can be made available to researchers interested in repeating these experiments.

Light from the two externally mounted LEDs was projected first through a neutral density filter (no. 298 0.15 ND, Lee filters, Burbank, CA, USA) if required to match overall intensity between the different filter sets (electronic supplementary material, table S1), then through a 0.28 mm (1/64 of an inch) thick sheet of Teflon that completely depolarized the light, followed by a thin film polarizer (no. 7300, Rosco, London, UK), and finally through one of our nine custom-made volume diffusers, before passing through the nematic LCD panel (figure 1). The 0% polarization setting was created by reversing the order of the combination of the filters such that the light passed through the Teflon last.

### (b) Part One: per cent polarization threshold test stimuli

Participants were asked to use their dominant eye (other eye was covered) to identify the orientation (horizontal or vertical) of a square wave grating made up of alternating bars with angles of polarization that were nearly vertical (96° from horizontal) and nearly horizontal (19° from horizontal). Previous research [28] has shown that Haidinger's brushes are more regular and less diffuse when observed with the dominant eye. To maintain the visual phenomenon of Haidinger's brushes, the angle of polarization in each bar was reversed every 0.5 s (schematic of stimuli and how they may appear to observer provided in electronic supplementary material, figure S4). Grating patterns were created and presented using PowerPoint (v. 2010, Microsoft, Redmond, WA, USA) with our modified LCD monitor connected to a PC as a secondary monitor displaying only the stimulus presentation (presenter mode). The square wave grating had a spatial frequency of 0.96 cycles per degree (see the electronic supplementary material for justification of spatial frequency).

### (c) Part One: per cent polarization threshold experimental set-up

The modified LCD monitor was positioned 55 cm in front of the participant, behind a black felt-lined box with holes cut in each end through which the participant viewed the stimulus. The two holes ensured that participants could only view the monitor at a viewing angle that was limited to 5.9° of arc, less than 3° from the normal to the plane of the LCD surface (see limitations of using modified LCD monitors in [29]). Fluorescent room lights remained on during the experiment; therefore, the felt-lined box had an extension that blocked direct light from overhead.

### (d) Part One: per cent polarization threshold task

We used a single interval binary choice paradigm in which participants had to identify the orientation of the stimulus grating pattern as horizontal or vertical. Nine levels of per cent polarization were used, ranging from 0 to 90% (electronic supplementary material, table S1). To find the threshold, we used the method of limits with two cycles of alternating ascent and descent, with three presentations of the stimulus at each level in a quasi-random order (12 presentations at each level; six horizontal and six vertical). Half of the participants began with an ascending cycle and half began with a descending cycle. We tested for potential errors owing to habituation or expectation by analysing the variance in mean thresholds for both ascending and descending runs.

Trials began with a computer-generated audible signal. Participants were given 3 s to observe before they could provide their answer. The 3 s delay was to encourage participants to consider each stimulus irrespective of the difficulty of the task in an attempt to minimize guessing. They were given 20 s before the stimulus disappeared and a second audible signal indicated the end of the trial. The next trial began after the participant stated their selection for the current stimulus. Verbal feedback on success rate was given after each set of three presentations to provide motivation. Short breaks were provided at the end of each ascending and descending run to reduce eye fatigue.

Because Haidinger's brushes are not immediately apparent to the untrained eye, participants underwent a short training or qualifying phase prior to participating in the experiment. Participants were required to correctly identify the orientation of five or more of six presentations with the per cent polarization set at 90%; if unsuccessful they did not carry on to the main experiment. Four out of 27 participants were rejected, reasons for which were not investigated.

### (e) Part One: data analysis

The participant responses were investigated with a general linear model (GLM, SPSS, v. 21, IBM, New York, NY, USA), with the variables: sex, order of testing (ascending or descending pass first), previous experience/knowledge of Haidinger's brushes and presence of contact lenses as fixed factors. Stepwise backwards elimination was conducted to achieve a minimal model, with effects removed sequentially in order of least significance.

To calculate the detection threshold, a psychometric function (logistic curve) was fit to the data using a nonlinear least-squares method (R, v. 3.1.2 [30]) with the probability of response, *y*, given by2.1
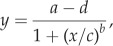
where *a* is the minimum point, *b* is the gradient at the point of inflection of the curve, *c* is the point of inflection, *d* is the maximum point, *y* is the predicted score (proportion correct) and *x* is the per cent polarization. For determining the threshold, we set the detection criterion to 75%, corresponding to nine out of 12 correct, which for a binomial response is statistically different from chance.

### (f) Part Two: polarized light production for testing rotational dynamics of Haidinger's brushes

A second identical LCD monitor was modified by removing only the front polarizer. As the per cent polarization was not varied in this part of the experiment, the original factory-installed light supply, back polarizer and diffuser of this unit were left intact. Photopic luminance at the viewing distance of 30 cm was 204 cd m^−2^. The degree of polarization was 98% and angle of polarization of the two settings used (RGB colour settings were 0, 0, 0 = black on a normal monitor and 255, 255, 255 = white on normal monitor) differed by 75°. Note that this angular difference was not identical to that of the per cent polarization system in which the additional filters added to the system altered the range of polarization angles attainable.

### (g) Part Two: rotational dynamics alignment task

**Figure 2.**
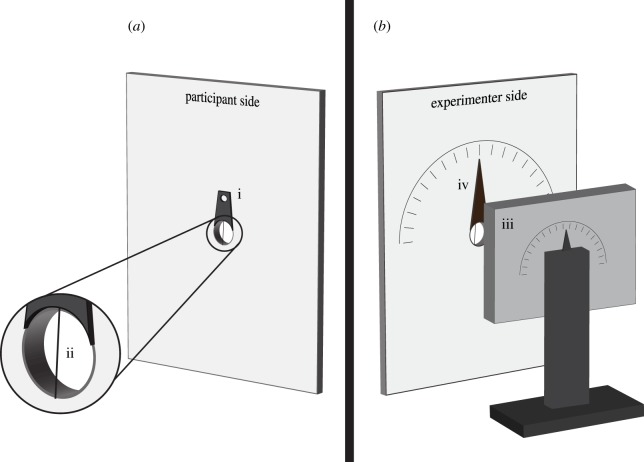
Experimental set-up for the alignment task designed to characterize the rotational dynamics of Haidinger's brushes. (*a*) Participants sat on one side of an opaque panel and used a handle (i) to rotate a thin piece of translucent monofilament fishing line (ii) until it was aligned with the long axis of the yellow component of Haidinger's brushes. (*b*) On the other side of the panel, the experimenter set the orientation of the modified LCD monitor (iii) that presented two alternating polarization orientations. One orientation was used to refresh Haidinger's brushes and the other was used for alignment; the latter was accompanied by an audible sound to inform the participant of which polarization orientation they should be aligning. The experimenter recorded the orientation set by the participant using the position of a pointer (iv), which was also attached to the rotating monofilament line holder (ii).

We designed an alignment task to measure how orientation of Haidinger's brushes varied as a function of polarization angle. Participants used their dominant eye for the task and their other eye was covered. Each participant aligned a piece of monofilament fishing line with the yellow axis of Haidinger's brushes when presented with a linearly polarized light field at a range of polarization angles, varied by rotating our modified LCD monitor (figure 2). The monofilament line bisected the aperture (58 mm diameter) of a rotatable photographic filter holder, with glass filter removed, which was mounted in the centre of an opaque screen. A handle on the participant's side of the screen was used to rotate the monofilament line to the desired orientation. The handle was also connected to a pointer on the experimenter's side of the screen that rotated over a large printed protractor (figure 2*b*), unseen by the participant. To maintain salience of Haidinger's brushes, the screen alternated between the two angles of polarization every 0.5 s. An audible tone accompanied the presentation of the stimulus polarization angle to which participants were to align.

The orientation of the incident polarized light field was controlled by rotating the LCD on a monitor stand and was measured with a second printed protractor mounted on the back of the LCD, visible only to the experimenter (figure 2*b*). Each participant made 36 alignments to 18 different orientations (two for every 10° from 0° to 170°) that were presented randomly (random numbers generated in Excel, v. 2010, Microsoft, which was simultaneously used to record participant alignment values). Participants informed the experimenter verbally when satisfied with their alignment, and no time constraints were placed on performing the task. Sessions typically required less than 30 min.

### (h) Part Two: data analysis

The rotational dynamics of Haidinger's brushes were analysed using an equation derived by Rothmayer *et al*. [25], which predicts the perceived orientation of Haidinger's brushes (*θ*_1_) as a function of incident polarization angle (*θ*_0_) and the retardation (Δ) introduced by the cornea. This approach assumes a uniaxial model of corneal birefringence. We modified Rothmayer *et al*.'s [25] equation to include an additional parameter, *s*, to account for the variable orientation of individual corneal azimuth values relative to *θ*_0_ = 0 in the experimental set-up2.2
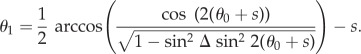


The data gathered from the alignment task were adjusted to correct for any systematic offset between presented and perceived polarization angles. The magnitude of the offset for each participant was determined by subtracting *θ*_0_ from *θ*_1_ and taking the average of this difference for all 36 alignments made by that participant. The expression above was fitted to each of the participant's normalized datasets using a nonlinear least-squares method in MATLAB Release 2014b, (The MathWorks, Inc., Natick, MA, USA). The fitted parameters Δ and *s* provided a measure of an individual's corneal retardance at 460 nm and the orientation of their corneal azimuth with respect to the experimental set-up.

## 3. Results

### (a) Per cent polarization threshold

**Figure 3.**
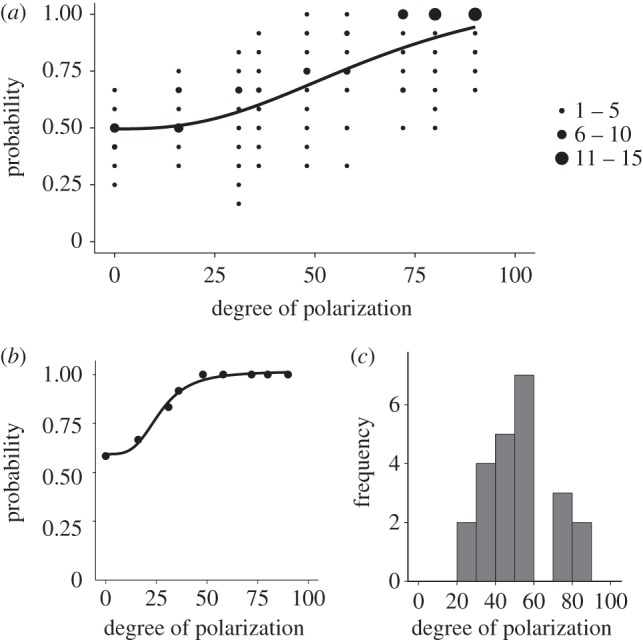
Per cent polarization threshold for humans with normal vision. (*a*) The mean (black curve) and variance (number of participants indicated by solid circle size; small = 1–5, medium = 6–10, large = 11–15) for the per cent polarization threshold curves of 23 participants performing a single interval binary choice experiment. The task involved correctly identifying the orientation of horizontal and vertical square wave gratings displayed in polarization-only contrast on a modified LCD (see §2b for details). The mean per cent polarization threshold (56%) corresponds to the stimulus setting at which the probability of successfully completing the task fell below 0.75. (*b*) One participant was able to continue to discriminate the gratings down to less than 25%. (*c*) The individual threshold values were normally distributed.

The average per cent polarization threshold was 56% ± 3% s.d. (figure 3*a*; *n* = 23), with individual thresholds as low as 23% (figure 3*b*) and extending upwards to 87% (figure 3*c*). Variation in participant thresholds was normally distributed; Shapiro–Wilk test of normality = 0.958, *p* = 0.429 (figure 3*c*). All of the following: wearing contact lenses (GLM, *F*_1,18_ = 0.190, *p* = 0.668); previous experience with/knowledge of Haidinger's brushes (GLM, *F*_1,19_ = 0.801, *p* = 0.382); sex (GLM, *F*_1,20_ = 3.201, *p* = 0.89); and the order of stimulus presentation either ascending or descending first pass (GLM, *F*_1,21_ = 2.068, *p* = 0.165) were not correlated to the per cent polarization thresholds measured.

### (b) Rotational dynamics of Haidinger's brushes

**Figure 4.**
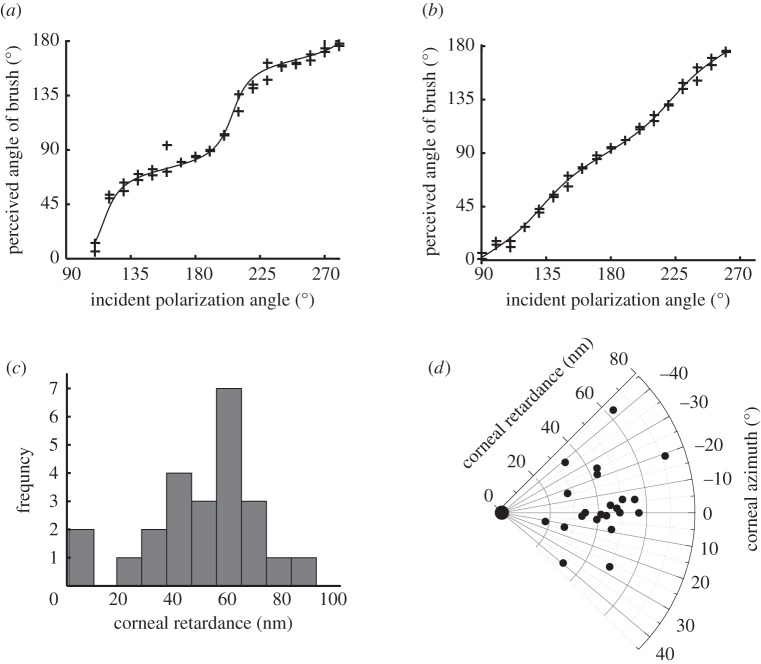
Nonlinear relationship between Haidinger's brushes (perceived polarization orientation) and the presented orientation of the polarized light field varied among individuals. In (*a*,*b*), each point is the reported orientation of the yellow axis of Haidinger's brushes relative to the orientation of the polarized stimulus produced by a modified LCD screen that was rotated around 180°. The solid line is the nonlinear least-squares regression fit to equation (2.2) provided in the text. (*a*) The individual with the strongest nonlinearity in perceived angle as a function of stimulus polarization angle orientation. This individual had the highest estimated corneal retardance in our study, 71.4° corresponding to 91.4 nm (*r*^2^ = 0.987). (*b*) An individual with an estimated retardance of 43.3°, which corresponds to 55.3 nm at 460 nm (*r*^2^ = 0.997). This is similar to the mean retardance value found in the population tested and shows only a slight nonlinearity. (*c*) Histogram of the corneal retardance values for 24 participants. (*d*) Distribution of the corneal retardance and corneal azimuth values for all participants; larger point at origin represents two individuals with zero retardance.

The relationship between orientation of Haidinger's brushes and the stimulus polarization orientation for each individual was typically nonlinear (figure 4*a*,*b*), and in some cases there was a rapid switching/change in orientation relative to the change in stimulus orientation (e.g. around 200° incident polarization angle in figure 4*a*). The values of corneal retardance ranged from 0° to a maximum of 72°, or 0.199*λ*, which corresponds to 91 nm at 460 nm (peak wavelength for spectral sensitivity curve of Haidinger's bushes [11]). The mean corneal retardance was 40° ± 17° s.d., corresponding to 51 ± 20 nm s.d. (figure 4*c*,*d*). Mean corneal azimuth was −4° ± 19° s.d. (nasally upwards; figure 4*d*). The mean offset was 83° ± 6° s.d. Three participants' datasets were rejected, as the fit to equation (2.2) gave *r*^2^ values lower than 0.4 indicating that the individuals were unable to perform the alignment task, the reasons for which were not investigated. All 21 remaining datasets had *r*^2^ values above 0.9.

## 4. Discussion

We found that humans can detect a visual stimulus in polarization-only contrast using Haidinger's brushes when per cent polarization is as low as 56%. In addition, we have characterized the rotational dynamics of Haidinger's brushes and verified the existence of the predicted nonlinearity between presented and perceived polarization orientations [25]. By quantifying this rotational ‘switching’ effect, we have developed a simple psychophysical method of estimating corneal retardance and corneal azimuth. These psychophysical tests were facilitated by adopting modified LCD technology recently used by researchers investigating polarization sensitivity in non-human animals [26,27,29,31–33].

While the mean per cent polarization threshold was 56% ± 3%, there was a normal continuous distribution of threshold values extending down to as low as 23%. This measured variance in per cent polarization threshold may be correlated with variation in the density or alignment of the zeaxanthin and lutein pigments in the macula (reviewed in [34]), which is thought to be the underlying mechanism mediating Haidinger's brushes. It is known that there is variability in macular pigment density (greater than fourfold differences in optical density) among individuals [35–37], and a comparison of macular pigment density with the dichroic ratio of the retina, using a psychophysical method that employed Haidinger's brushes, showed a ‘very close agreement’ [11]. The potential for a close correlation between an individual's per cent threshold for detecting Haidinger's brushes and their macular pigment density could make perception of polarization a useful tool in the assessment of retinal pathology in which low macular pigment density is a risk factor, or where there is progressive deterioration of the macula or Henle fibre layer.

An experiment that used decreasing intensity, rather than decreasing per cent polarization, found that subjects with various ocular diseases were poorer at detecting Haidinger's brushes than subjects with normal vision at low light intensities [5]. Thus, Haidinger's brushes could be used to detect retinal dysfunction, but, given the wide range of diseases detected, would not be valuable for differential diagnosis of existing disease. However, people with low macular pigment density are at greater risk of acquiring AMD [16,38], and therefore the ability to quickly and easily measure macular pigment density using per cent polarization threshold could be a useful tool for identifying those at risk and tracking changes in the health of the macula. Potential advantages of the method are that a small user-controlled version of our testing apparatus could easily be devised for self-assessment. Moreover, this method provides additional information about the spatial ordering of the macular pigment with respect to the Henle fibre layer when compared with traditional heterochromatic flicker photometric measures of macular pigment density. Whether a loss of the orientational order of the macular pigment occurs in early stages of AMD and what the clinical implications are, have yet to be addressed.

In the second part of this study, we characterized the rotational dynamics of Haidinger's brushes and demonstrated a nonlinear relationship between the presented and perceived angles of polarization for some individuals, confirming the prediction of Rothmayer *et al*. [25]. The values of corneal retardance derived from this test cover a range from 0 nm to 91 nm with a mean of 51 nm, in close agreement with the retardance derived from psychophysical data on apparent retinal dichroic ratio (68 nm) [6] and Mueller matrix ellipsometry (39–86 nm) [39]. Similar ranges have been determined by larger-scale studies of the adult Caucasian population using scanning laser polarimetry with variable corneal compensation (7–91 nm) [40] and observation of the fourth Purkinje image (0–125 nm) [22]. These ranges imply individual differences in the orientation of the birefringent fibre layers and/or the number of layers (i.e. thickness) of the corneal stroma. Individuals with highly retarding corneas, who perceive a rotational nonlinearity, would also be expected to perceive Haidinger's brushes generated by circularly polarized light as being of higher contrast than individuals with weakly retarding corneas. The large variability in corneal retardance observed here is therefore consistent with the variability in reports of Haidinger's brushes perceived in circularly polarized light [3,24,41,42].

Corneal azimuth was also found to vary considerably among individuals (figure 4), with a mean value of −4° ± 19° (nasally upwards). Other studies of polarization axes in adult Caucasians have found polarization (slow) axes oriented approximately 20° nasally downwards, with a large range (−54° to 90°) [40,43]. Our method does not distinguish between the fast and slow axes, and therefore the reported range is restricted to ±45°. This restriction, coupled with the low age range of our, predominantly student, sample may explain the discrepancy in the mean values, as younger adults have been shown to have corneal polarization axes oriented more strongly nasally downward [40] and over 45° this would correspond to low nasally upward values in our frame of reference. The large variation in corneal azimuth measured by this study and others has implications for retinal scanning and intraocular assessment technologies that use polarized light fields in their measurements [44].

Linearly polarized light transmitted through a birefringent cornea will, unless aligned with the fast or slow axis, become elliptically polarized. The overall effect of corneal birefringence is therefore to modulate the apparent contrast of Haidinger's brushes as a function of angle [6,25,44]. Based on the mean retardance value observed in this study (51 nm), Haidinger's brushes are predicted to decrease to a minimum of 64% of full contrast, at 45° from the corneal azimuth. For the individual with the highest corneal retardance in this study, the contrast of a brush oriented at 45° from the corneal azimuth is predicted to fall to 32% of the maximum [25]. Consideration of this effect is therefore merited in the design of future experiments testing the relationship between macular pigment density and polarization sensitivity threshold for linearly polarized light.

The model proposed by Rothmayer *et al.* [25], and used to fit the rotational dynamics data in this study, assumes a uniaxial model of corneal birefringence. While this has been shown to be a suitable approximation for light at perpendicular incidence on the central cornea [39], in general the cornea is understood to behave as a biaxial crystal, with retardance varying as a function of position [39,45]. It would be interesting to explore what, if any, perceptual effects would be predicted by a full biaxial model, for example a change in the perceived rotational nonlinearity as a function of pupil dilation.

It has been well established that many animals use polarization information for a variety of tasks and, unsurprisingly, some species have evolved highly sensitive polarization detection systems [1,27,46]. Apart from humans, the only other vertebrate to be tested at different per cent polarization levels has been rainbow trout (*Oncorhynchus mykiss*), which was found under laboratory conditions to require polarization in excess of 65–75% to perform an orientation task [47,48] and 63–72% to perform a predation task [49]. Invertebrates are more sensitive (see electronic supplementary material, table S2, for a list of all species tested to date) with some able to respond to polarization-only contrast, when the per cent polarization is as low as 6–7% [31,49]. The lower sensitivity of vertebrates compared with invertebrates might be interpreted as an indication that vertebrates as a whole are not well adapted to using polarized light, especially considering the often low levels of polarization present in the natural environment, where for example underwater polarization rarely exceeds 50% [50] and celestial polarization rarely exceeds 80%. Previous experiments using modified LCDs to test polarization vision in seals and fishes failed to show behavioural responses [26,33], even in fishes previously reported to be polarization sensitive [51–53]. To explain the lack of innate responses in fishes, it was suggested that these animals do not use polarization information in the context tested and/or that they do not use polarization for image parsing. Alternatively, it could be that, like humans, they see polarization as a subtle phenomenon, and that training is required to test discrimination [54]. In the context of animal behaviour, the only function that could be mediated by the human capacity to detect polarization would be the ability to detect the position of the sun in the sky using the celestial polarization pattern, which could conceivably be used as a navigational aid. Our results suggest that this would only be possible when the degree of polarization was above an individual's per cent polarization threshold, which based on measurements of sky polarization would limit this to conditions of clear skies [55,56]. There is some evidence that Vikings may have used celestial polarization patterns as a navigational aid when crossing the north Atlantic [57–59]. Celestial polarization patterns could have been particularly helpful in northern latitudes where the twilight period, when the sun is below the horizon and the sky is too bright to see the stars, is particularly long [60].

## 5. Conclusion

Haidinger's brushes give humans, on average, the ability to detect the orientation of a polarized light field even when the per cent polarization is as low as 56%. We have also demonstrated the existence of the predicted mismatch between the actual and perceived polarization angle [25]. By quantifying the precise rotational dynamics of Haidinger's brushes, we have developed a psychophysical method of estimating corneal retardance and azimuth. The mechanism underlying human sensitivity to polarization is better understood than it is for most other vertebrates, and the role of macular pigments and the Henle fibre layer means that it may be possible to use modified versions of our tests as a tool for detecting susceptibility to AMD, which has been linked to pre-existing low macular pigment densities [16,61]. The technology used in these experiments could potentially be developed into a small affordable device for self-assessment in clinic waiting rooms.

## Supplementary Material

Supplemental Material

## Endnote

1 Throughout this work, we will use the term polarized light to refer to linearly polarized light and will directly state when we are referring to circular or elliptical polarization.
