# Supplementary material for: Perceiving polarization with the naked eye: characterization of human polarization sensitivity
Source: Proc Biol Sci. 2015 Jul 22;282(1811):20150338. doi: 10.1098/rspb.2015.0338 (PMC4528539; doi:10.1098/rspb.2015.0338)
Supplement: Supplemental Material [file rspb20150338supp1.docx]

**Supplemental material**

**
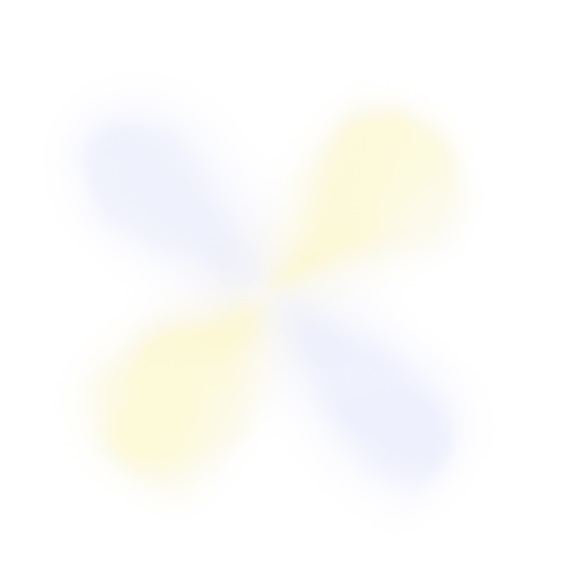
Figure S1**. Schematic of Haidinger’s brushes as it is perceived by the authors. Note the colours are intentionally made stronger here than they actually appear so that the effect is visible when reproduced online or in print.


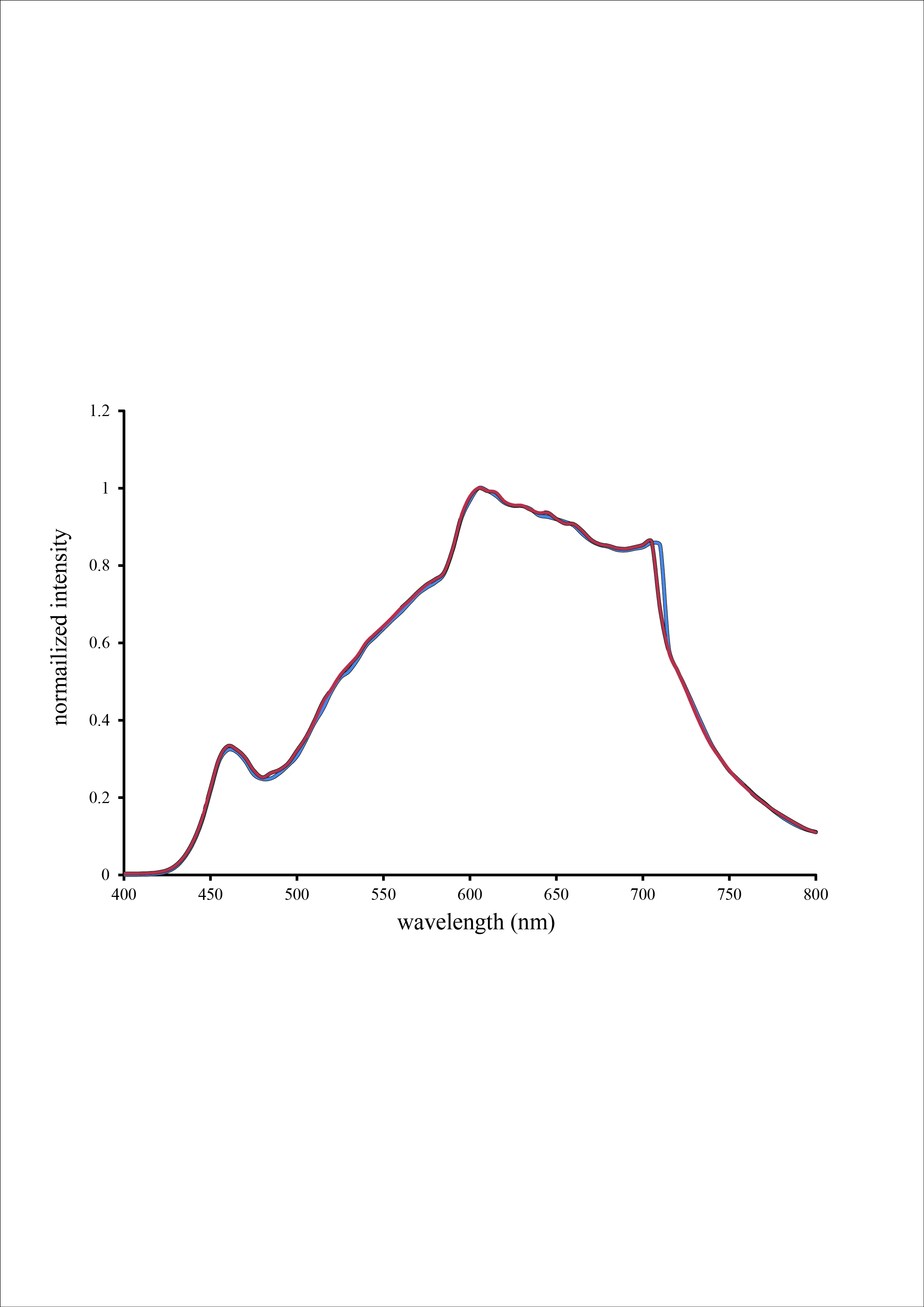


**Figure S2**. Example of spectral difference between two polarization states used in the stimulus gratings. We present the curves for our 90 per cent polarization stimulus, which had the highest just noticeable difference value (0.204) between the bars of the polarized grating.

**
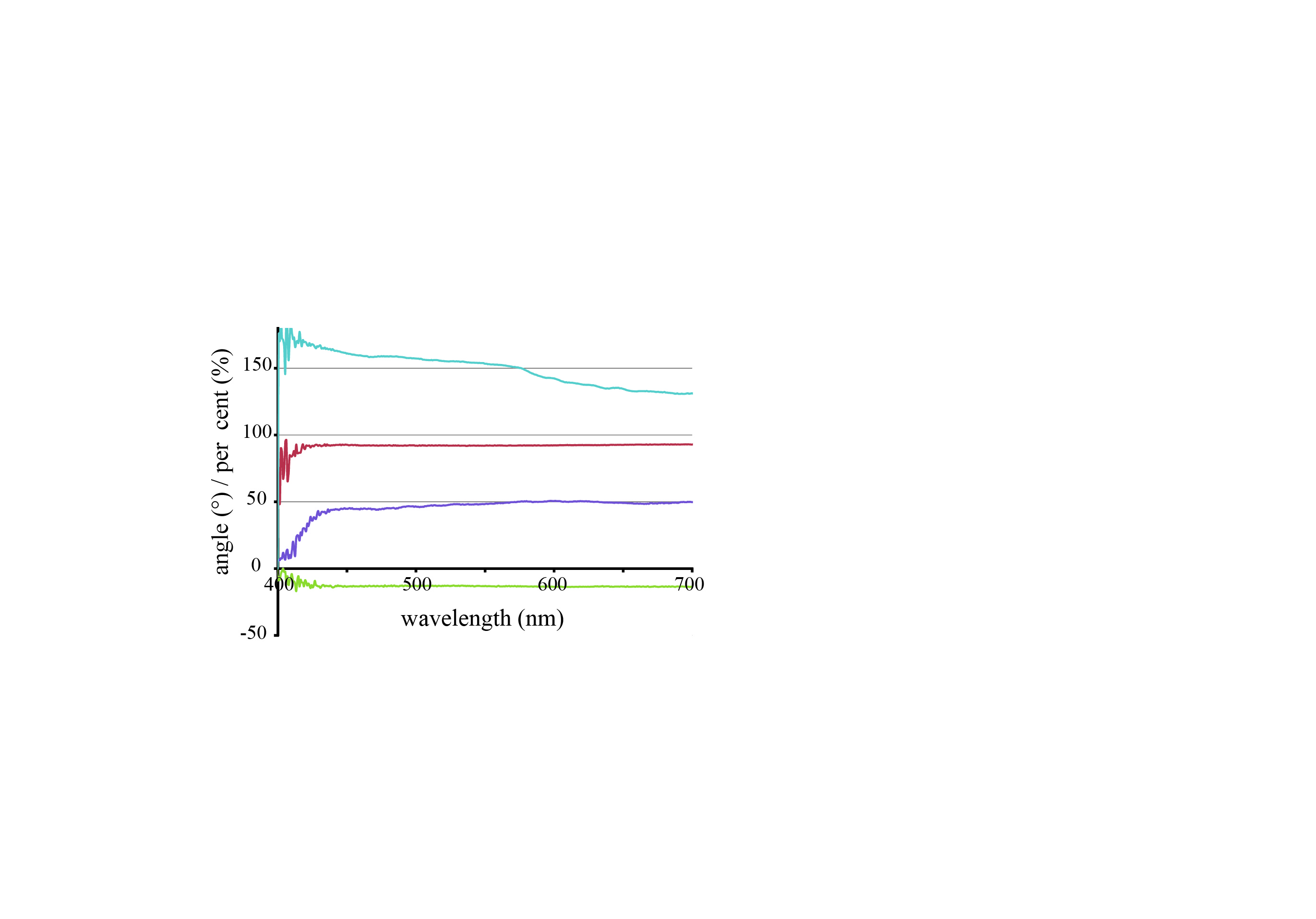
**

**Figure S3**. Representative plots of per cent polarization and angle as a function of wavelength (a) for the modified polarization-liquid crystal display monitor with a per cent polarization volume diffuser. The uppermost (blue) line shows the polarization angle when the monitor was set to white (255, 255, 255), the second line from the top (red) shows the polarization angle when the monitor was set to black (0, 0, 0), the third line from the top (purple) shows the per cent polarization, and the lowest (green) line shows the ellipticity, each as a function of wavelength.

**Determination of spatial frequency for polarization-only contrast gratings**

Preliminary experiments were done to determine the most salient spatial frequency for the detecting gratings presented in polarization-only contrast using the human perception of Haidinger’s brushes. Participants found the task easiest when spatial frequency was 0.96 cycles per degree. This value does not match the typical human contrast sensitivity function for the intensity used here (200 cd m^-2^ ≈ 10000 Trolands for 8 mm pupil diameter; but see statement in next section about pupil diameter), which would be expected to peak between 2 and 10 cycles per degree for achromatic contrast. However, contrast sensitivity for coloured gratings (*e.g*. blue-yellow as Haidinger’s brushes are perceived) is lower than for achromatic gratings at the same luminance [1], which may explain the requirement for a lower spatial frequency.

**Calculation of intensity contrast across the spectrum**

When using modified LCD screens to present stimuli in polarization-only contrast, the experimental methodology relies on there being no detectable intensity contrast associated with different presented angles of polarization. We measured the contrast produced by our system under every condition used and compared these to existing human contrast modulation thresholds (citations below). We calculated both Michaelson contrast, which is typically used for gratings, and Weber contrast, which is typically used for small sharp edged objects against a uniform background, because, while our pattern was a grating, the appearance of Haidinger’s brushes, and particularly the central cross region, could be perceived as a small object set in a uniform background. Given that there is no precedent in literature, and it is not clear which contrast function would be most relevant in such a scenario, we have taken the more conservative case of Weber contrast. The maximum intensity contrast produced by our modified LCD, after multiplying our measured contrast by 4/π, as per [2] to account for the fact that we used square wave gratings and not sine wave gratings, had a Weber contrast of 0.45%. The limit of contrast detection at ~200 cd m^-2^ or 10000 Trolands (intensity of our system at the eye of a subject with an 8 mm pupil) at a spatial frequency of 1 cycle per degree is approximately 1% Weber contrast [3], therefore all of our stimuli (Table S1) are below the limit of human contrast detection at any illumination and spatial frequency [3]. The value of 10000 Trolands is conservative because we have assumed a pupil diameter of 8 mm. In reality, pupil diameters at 200 cd m^-2^ would be closer to 5.5 mm for 20-30 year olds (which make up the bulk of our participants), and smaller still for older participants [4], this would mean that the actual intensity was probably less than 5000 Trolands a value which would increase the threshold for intensity contrast detection above 1%.

To ensure that there was no chromatic cues that could be detected we calculated the just noticeable difference values for each of our per cent polarization settings using the receptor noise limited model of Vorobyev and Osorio [5]. Weber fractions were set to 0.018 for l-cones, 0.019 for m-cones and 0.087 the s-cone as per [6]. The just noticeable difference values for our gratings did not exceed 0.3, which is well below the detection limit [7], and actual spectral intensity differed little from 400-700 nm (figure S2).

**Table S1**. Characterization of the per cent polarization filters mounted on our modified polarization-liquid crystal display monitor. Per cent polarization was measured with a Glan-Thompson polarizer and Fresnel Rhomb prism combination mounted at the end of a fibre optic cable connected to a spectrophotometer (QE 65000, Ocean Optics, Dunedin, FL, USA) following the procedure described in the supplemental material provided in [8].

| **Per cent  polarization** | **ND filter present** | **LCD  setting (white or black)** | **Photopic  luminance (cd/m^2^)** | **Photopic  luminance for 8 mm pupil (Trolands)** | **Michaelson’s contrast (%)** | **Corrected* Michaelson's  contrast (%)** | **Corrected*  Weber contrast (%)** | **Just Noticeable Difference✝**  **(no units)** |
| --- | --- | --- | --- | --- | --- | --- | --- | --- |
| 90 | 0.15 | w | 170.15 | 8553.50 | 0.102 | 0.130 | 0.259* | 0.204 |
|  |  | b | 170.50 | 8570.94 |  |  |  |  |
| 80 | 0.15 | w | 168.85 | 8488.28 | 0.097 | 0.123 | 0.247 | 0.153 |
|  |  | b | 169.18 | 8504.75 |  |  |  |  |
| 72 | 0.15 | w | 152.67 | 7674.86 | 0.105 | 0.134 | 0.268* | 0.013 |
|  |  | b | 152.99 | 7691.02 |  |  |  |  |
| 58 | 0 | w | 183.01 | 9199.72 | 0.081 | 0.103 | 0.206 | 0.124 |
|  |  | b | 183.30 | 9214.66 |  |  |  |  |
| 48 | 0 | w | 187.99 | 9450.24 | 0.031 | 0.039 | 0.079 | 0.094 |
|  |  | b | 188.11 | 9456.07 |  |  |  |  |
| 36 | 0 | w | 156.06 | 7845.38 | 0.178 | 0.226 | 0.452* | 0.043 |
|  |  | b | 155.51 | 7817.56 |  |  |  |  |
| 32 | 0 | w | 165.44 | 8316.54 | 0.128 | 0.163 | 0.325* | 0.059 |
|  |  | b | 165.86 | 8337.81 |  |  |  |  |
| 16 | 0 | w | 157.92 | 7938.87 | 0.037 | 0.048 | 0.095 | 0.020 |
|  |  | b | 158.04 | 7944.81 |  |  |  |  |
| 0 | 0 | w | 180.51 | 9074.01 | 0.019 | 0.024 | 0.049 | 0.008 |
|  |  | b | 180.44 | 9070.53 |  |  |  |  |

* Four of the filter sets resulted in corrected Weber contrast greater than 0.25%, which is still below detection threshold at 10000 Trolands [3]. This is confirmed by there being no increase in probability of correct choice at these specific values (see figure 3a in main text).

**✝** Just noticeable differences were calculated using the Vorobyev Osorio receptor noise limited model [5] with the Weber fractions set to 0.087 for s-cone, 0.019 for m-cone and 0.018 for l-cone as per [6]. Just noticeable difference values less than 1.0 are not detectable.

Table S2. List of species for which the per cent polarization threshold has been measured.

| **Animal** | **Scientific name** | **Per cent polarization threshold** | **Polarization angle contrast** | **Citation** |
| --- | --- | --- | --- | --- |
| Human | *Homo sapiens* | 56% (mean)  24% (lowest recorded) | 65° | This work |
| Rainbow trout | *Oncorhynchus mykiss* | 63-75% | N/A* | Hawryshyn and Bolger 1990 [9]  Novales Flamarique and Browman 2001 [10] |
| Marine copepod | *Pontella karachiensis* | 20-30% | N/A* | Manor *et al*. 2009 [11] |
| Honey bee | *Apis melifera* | 10% | N/A* | Edrich and von Helversen 1976 [12] |
| Freshwater crayfish | *Procambrus clarki* | 6-13% | 20° | Glantz and Schroeter 2007 [13] |
| Field cricket | *Gryllus compestris* | 7% | N/A* | Henze and Labhart 2007 [14] |

*In these cases the task was a navigation task and did not involve the comparison of two e-vectors.

**
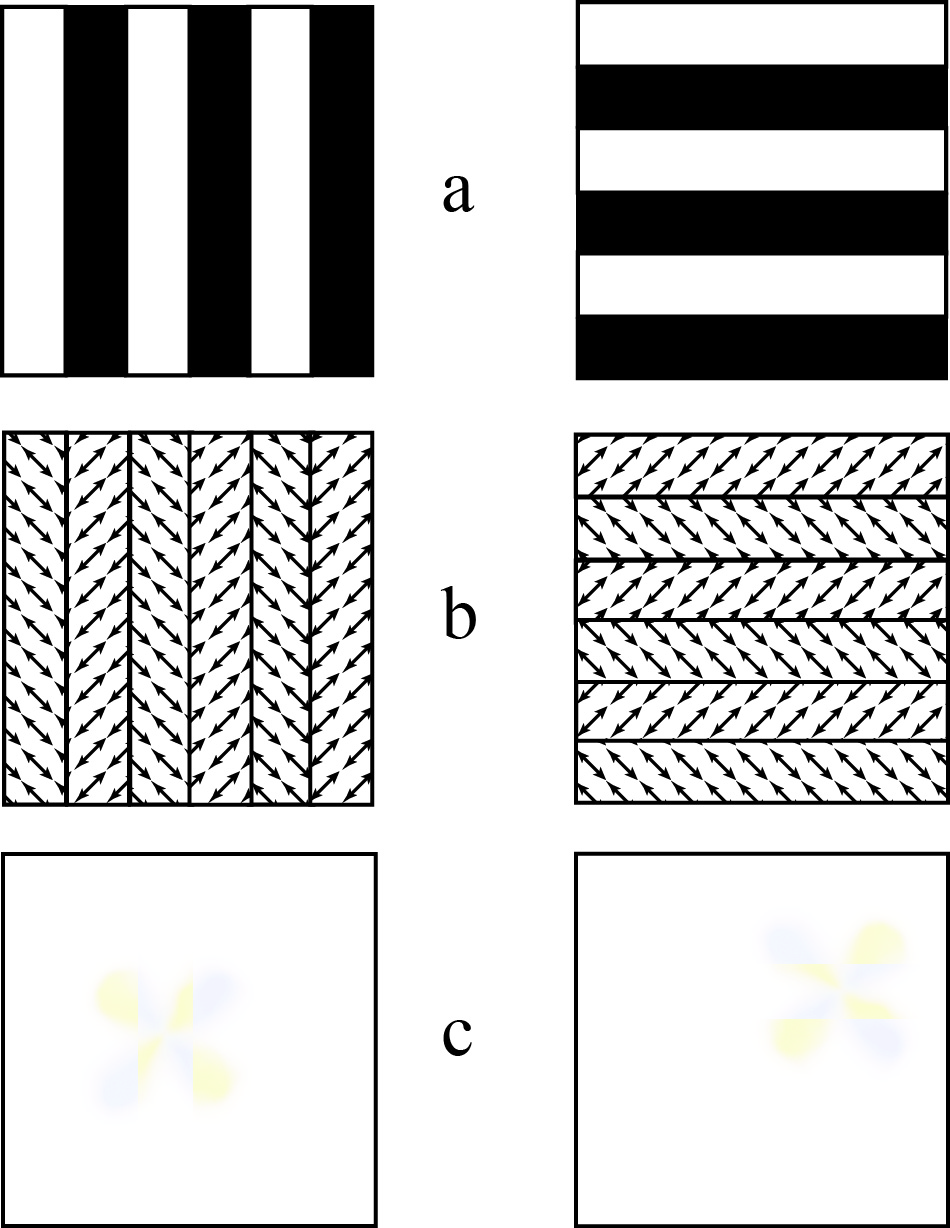
**

**Figure S4**. Schematic of stimuli used in the experiment, showing: how the stimuli would appear if it were presented on a normal, unmodified, LCD monitor (a); the orientation of the polarization stimuli generated by these patterns when displayed on a modified LCD monitor (b) and; a representation of how Haidinger’s brushes generated by these polarization stimuli appear to author SET (c) fixating at the center of the field of view (left) of the vertical bar stimulus and toward the top (right) of the horizontal bar stimulus. Haidinger’s brushes are altered at the edges of the bars where contrasting polarization angles are in close apposition, and this is what allows the observer to determine the orientation of the grating. As in Figure S1 the colours in panel (c) are intentionally made stronger to that the effect is visible when reproduced online or in print.

**Literature cited in supplemental material**

[1] Mullen, K.T. 1985 The contrast sensitivity of human color vision to red-green and blue-yellow chromatic gratings. *J. Physiol.* **359**, 381-400.

[2] Campbell, F.W. & Robson, J.G. 1968 Application of fourier analysis to visibility of gratings. *J. Physiol.* **197**, 551-&.

[3] Van Nes, F.L. & Bouman, M.A. 1967 Spatial modulation transfer in the human eye. *J. Opt. Soc. Am.* **57**, 401-406.

[4] Winn, B., Whitaker, D., Elliott, D.B. & Phillips, N.J. 1994 Factors affecting light-adapted pupil size in normal human subjects. *Invest. Ophthalmol. Vis. Sci.* **35**, 1132-1137.

[5] Vorobyev, M. & Osorio, D. 1998 Receptor noise as a determinant of colour thresholds. *P. Roy. Soc. B Biol. Sci.* **265**, 351-358.

[6] Stiles, W.S. 1959 Color vision: The approach through increment threshold sensitivity. *Proc. Natl. Acad. Sci. U.S.A.* **45**, 100-114. (doi:DOI 10.1073/pnas.45.1.100).

[7] Olsson, P., Lind, O. & Kelber, A. 2015 Bird colour vision: behavioural thresholds reveal receptor noise. *J. Exp. Biol.* **218**, 184-193. (doi:10.1242/jeb.111187).

[8] Temple, S.E., Pignatelli, V., Cook, T., How, M.J., Chiou, T.-H., Roberts, N.W. & Marshall, N.J. 2012 High-resolution polarisation vision in a cuttlefish. *Curr. Biol.* **22**, R121-122. (doi:10.1016/j.cub.2012.01.010).

[9] Hawryshyn, C.W. & Bolger, A.E. 1990 Spatial orientation of trout to partially polarized light. *J. Comp. Physiol. A Neuroethol. Sens. Neural Behav. Physiol.* **167**, 691-697.

[10] Novales Flamarique, I. & Browman, H. 2001 Foraging and prey-search behaviour of small juvenile rainbow trout (*Oncorhynchus mykiss*) under polarized light. *J. Exp. Biol.* **204**, 2415-2422.

[11] Manor, S., Polak, O., Saidel, W.M., Goulet, T.L. & Shashar, N. 2009 Light intensity mediated polarotaxis in *Pontella karachiensis*. *Vision Res.* **49**, 2371-2378.

[12] Edrich, W. & von Helversen, O. 1976 Polarized light orientation of honey bee: the minimum visual angle. *J. Comp. Physiol.* **109**, 309-314.

[13] Glantz, R.M. & Schroeter, J.P. 2007 Orientation by polarized light in the crayfish dorsal light reflex: behavioral and neurophysiological studies. *J. Comp. Physiol. A Neuroethol. Sens. Neural Behav. Physiol.* **193**, 371-384. (doi:10.1007/s00359-006-0191-9).

[14] Henze, M.J. & Labhart, T. 2007 Haze, clouds and limited sky visibility: polarotactic orientation of crickets under difficult stimulus conditions. *J. Exp. Biol.* **210**, 3266-3276. (doi:10.1242/Jeb.007831).

**Raw Data**

| **Participant** | **Gender (Male= 0, Female= 1)** | **Experience (Yes=1, No=0)** | **Contact Lenses (Yes=1, No=0)** | **Threshold** | **Ascending (1), Descending (0)** | **Red filter (N=0, Y=1)** | **Pass 1 Number Correct** | **Pass 2 Number Correct** | **Pass 3 Number Correct** | **Pass 4 Number Correct** | **Horizontal Correct** | **Vertical Correct** | **retardance recalculated  by Juliette Nov 2014** | **Corneal Angle** | **Corneal Angle Adjusted 161** | **Corneal Angle Adjusted 96** | **Predicted Contrast White** | **Predicted Contrast Black** | **Cornea R^2** |
| --- | --- | --- | --- | --- | --- | --- | --- | --- | --- | --- | --- | --- | --- | --- | --- | --- | --- | --- | --- |
| 1 | 0 | 1 | 0 | 44.69 | 1 | 0 | 17 | 24 | 20 | 21 | 38 | 44 | 42.53 | -76.69 | -5.69 | -70.69 | 0.38 | 1.00 | 0.95 |
| 2 | 0 | 1 | 0 | 50.33 | 1 | 0 | 18 | 19 | 19 | 20 | 37 | 39 | 49.89 | -89.33 | -18.33 | -83.33 | 0.95 | 1.00 | 0.96 |
| 3 | 0 | 1 | 0 | N/A | N/A | N/A | N/A | N/A | N/A | N/A | N/A | N/A |  | -80.17 | -9.17 | -74.17 | 1.00 | 1.00 | 0.03 |
| 4 | 1 | 0 | 0 | 58.00 | 1 | 0 | 21 | 16 | 13 | 17 | 41 | 29 | 33.20 | -100.33 | -29.33 | -94.33 | 0.95 | 1.00 | 0.35 |
| 5 | 0 | 1 | 0 | N/A | N/A | N/A | N/A | N/A | N/A | N/A | N/A | N/A |  | -78.86 | -7.86 | -72.86 | 1.00 | 0.98 | -0.05 |
| 6 | 1 | 0 | 1 | 70.62 | 0 | 0 | 15 | 19 | 16 | 19 | 33 | 36 | 39.42 | -88.06 | -17.06 | -82.06 | 0.95 | 0.86 | 0.94 |
| 7 | 0 | 0 | 0 | 48.00 | 1 | 0 | 19 | 22 | 15 | 20 | 43 | 33 | 45.88 | -87.25 | -16.25 | -81.25 | 0.55 | 0.70 | 0.98 |
| 8 | 1 | 0 | 1 | 39.10 | 1 | 0 | 20 | 18 | 21 | 23 | 41 | 41 | 41.07 | -78.89 | -7.89 | -72.89 | 1.00 | 1.00 | 0.84 |
| 9 | 1 | 0 | 0 | 37.70 | 0 | 0 | 21 | 24 | 19 | 19 | 41 | 42 | 56.70 | -85.72 | -14.72 | -79.72 | 0.50 | 0.75 | 0.93 |
| 10 | 0 | 0 | 0 | 51.11 | 0 | 0 | 17 | 16 | 19 | 21 | 40 | 33 | 34.48 | -65.25 | 5.75 | -59.25 | 0.62 | 0.72 | 0.19 |
| 11 | 0 | 0 | 0 | N/A | 0 | 0 | 19 | 15 | 16 | 16 | 35 | 31 | 50.23 | -94.42 | -23.42 | -88.42 | 0.97 | 0.70 | 0.96 |
| 12 | 0 | 0 | 1 | 35.60 | 0 | 1 | 22 | 23 | 21 | 23 | 45 | 44 | 26.53 | -90.75 | -19.75 | -84.75 | 0.62 | 0.99 | 0.91 |
| 13 | 1 | 0 | 0 | 72.92 | 0 | 0 | 21 | 16 | 18 | 16 | 43 | 28 | 32.78 | -80.28 | -9.28 | -74.28 | 0.96 | 0.66 | 0.95 |
| 14 | 0 | 0 | 0 | 70.58 | 1 | 0 | 14 | 16 | 18 | 21 | 39 | 30 | 62.61 | -92.61 | -21.61 | -86.61 | 1.00 | 1.00 | 0.85 |
| 15 | 1 | 1 | 0 | 33.79 | 0 | 0 | 22 | 22 | 20 | 23 | 42 | 44 | 28.36 | -80.31 | -9.31 | -74.31 | 1.00 | 0.99 | 0.94 |
| 16 | 1 | 0 | 0 | 47.95 | 0 | 0 | 22 | 20 | 20 | 22 | 43 | 41 | 47.58 | -83.92 | -12.92 | -77.92 | 1.00 | 1.00 | 0.91 |
| 17 | 1 | 1 | 0 | 86.90 | 1 | 0 | 11 | 20 | 13 | 15 | 27 | 32 | 18.35 | -75.11 | -4.11 | -69.11 | 0.99 | 1.00 | 0.95 |
| 18 | 1 | 1 | 0 | 43.46 | 1 | 0 | 22 | 21 | 19 | 20 | 43 | 39 | 0.00 | -75.28 | -4.28 | -69.28 | 1.00 | 1.00 | 0.89 |
| 19 | 1 | 0 | 0 | N/A | N/A | N/A | N/A | N/A | N/A | N/A | N/A | N/A |  | -77.83 | -6.83 | -71.83 | 1.00 | 1.00 | 0.77 |
| 20 | 0 | 0 | 0 | 45.57 | 1 | 1 | 20 | 21 | 22 | 18 | 38 | 43 | 48.83 | -83.14 | -12.14 | -77.14 | 0.94 | 0.99 | 0.95 |
| 21 | 1 | 0 | 0 | 65.51 | 0 | 0 | 23 | 19 | 19 | 20 | 37 | 44 | 43.31 | -86.81 | -15.81 | -80.81 | 0.99 | 0.77 | 0.99 |
| 22 | 1 | 0 | 0 | 74.98 | 0 | 0 | 16 | 20 | 15 | 13 | 31 | 33 | 55.24 | -75.39 | -4.39 | -69.39 | 0.99 | 0.99 | 0.79 |
| 23 | 1 | 0 | 1 | 82.61 | 1 | 0 | 13 | 16 | 12 | 14 | 24 | 31 | 0.00 | -78.06 | -7.06 | -72.06 | 1.00 | 1.00 | 0.93 |
| 24 | 0 | 1 | 0 | 23.80 | 0 | 1 | 26 | 23 | 24 | 23 | 47 | 49 | 33.43 | -82.56 | -11.56 | -76.56 | 0.44 | 0.68 | 0.96 |
| 25 | 0 | 0 | 0 | 59.40 | 1 | 0 | 20 | 17 | 24 | 16 | 26 | 51 | 45.04 | -76.58 | -5.58 | -70.58 | 0.88 | 1.00 | 0.88 |
| 26 | 1 | 1 | 1 | 55.01 | 0 | 0 | 19 | 19 | 21 | 19 | 43 | 35 | 71.53 | -85.97 | -14.97 | -79.97 | 0.84 | 0.99 | 0.97 |
| 27 | 1 | 1 | 0 | 52.78 | 1 | 0 | 17 | 21 | 18 | 17 | 40 | 33 | 43.48 | -82.11 | -11.11 | -76.11 | 1.00 | 0.98 | 0.71 |
